# Supplementary material for: Genetic Diversity and Population Structure in Türkiye Bread Wheat Genotypes Revealed by Simple Sequence Repeats (SSR) Markers
Source: Genes (Basel). 2023 May 29;14(6):1182. doi: 10.3390/genes14061182 (PMC10298624; doi:10.3390/genes14061182)
Supplement: Supplementary file 1 [file genes-14-01182-s001.zip › genes-2406776-supplementary.pdf]

genes MDPI

Table S1. Details of 120 SSR markers, including primer name, primer sequences and chromosomal location.

Primer No.	Marker Name	Forward (5′–3′)	Backward (5′–3′)	Chromosomal Location
1	BARC 1	TTCCCTGTGTCTTTCTAATTTTTT	GCGAACTCCCGAACATTTTTAT	A5
2	BARC 3	TTCCCTGTGTCTTTCTAATTTT	GCGAACTCCCGAACATTTTTAT	6A
3	BARC 24	CAGCGCTCCCCGACTCAGATCCTT	GCGCCATGTTTCTTTTATTACTCACT	B6
4	BARC 37	GCCGCCTACCACAGAGTTGCAGC	GCGGCATTGACAAGACCATAGC	A6
5	BARC 45	CCCAGATGCAATGAAACCACA	GCGTAGAACTGAAGCGTAAAATT	3A, 2B
6	BARC 48	GCGAACAGGAG	GCGCTTTCCCACGTTCCATGTTTC	A6-B6
7	BARC 54	GCGTTGGCTAATCATCGTTCCTTC	AGCACCCTACCCAGCGTCAGTCAA	D6-A3
8	BARC 59	GCG GAG TCT GCA AT	GCA TCC ACC TCC GCA GTC AGT	B5-D2
9	BARC 64	CGCGATCGATCTCCCGGTTTGCT	GGGAAGAGGACCAAGGCCACTA	A7
10	BARC 73	AGGGTTACAGTTTGCTCTTTTAC	CCCGACGACCTATCTATACTTCTCT	B3
11	BARC 78	GCGAATTAGCATCTGCATCTGTTT	CGGTCAACCAACTACTGCACAAC	A4
12	BARC 80	GCTCACCGGGCATTGGGATCA	GCGATGACGAGATAAAGGTGGAG	B1
13	BARC 88	GGGCGCGGCACCAGCACTACC	GGGCGCGGCACCAGCACTACC 3'	B5
14	BARC 89	GCGGTTGTGATGTGCTGAAAGAT	GCGTGGGCTGTTTCTTCCTTTTGTT	B5
15	BARC 94	GGGGTGTGGTTGTTTGTAAGG	TGCGAATTCTATATACGATCTTGA	B7-A5
16	BARC 101	GCTCCTCTCACGATCACGCAAAG	GCGAGTCGATCACACTATGAGCCAATG	B3
17	BARC 105	CAGGAAGAAAAGGAAAGCATG	GCGGTGTGGCAATAATTACTTTTT	7A-4D
18	BARC 113	TCATGCGTGCTAAGTGCTAA	GAGGGCAGGAAAAAGTGACT	A6-A3
19	BARC 122	CCCGTGTATATCCAGGAGTG	CAGCCCTTGTGATGTGATG	5AL12, 2A
20	BARC 128	GCGGGTAGCATTTATGTTGA	CAAACCAGGCAAGAGTCTGA	B1-B2
21	BARC 130	CGGCTAGTAGTTGGAGTGTTGG	ACCGCCTCTAGTTATTGCTCTC	D5
22	BARC 133	AGCGCTCGAAAAGTCAG	GGCAGGTCCAACTCCAG	5DL-3BS
23	BARC 135	ATC GCC ATC TCC TCT ACC A	GCG AAC CCA TGT GCT AAG T	A5- D6
24	BARC 140	CGCCAACACCTACCATT	TTCTCCGCACTCACAAAC	B1-B2-B6
25	BARC 141	GGCCCATGGATAATTTTTGAAATG	CAATTCGGCCAAAGAAGAAGTCA	A5-B3-D5
26	BARC 142	CCGGTGAGAGGACTAAAA	GGCCTGTCAATTATGAGC	5BL,2D, 5A
27	BARC 152	CTTCCTAAAATCGGGCAACCGCTT-GTTG	GCGTAATGATGGGAGTGGCTATAGGG-CAGTT	B5-D5-B2- B1- D1
28	BARC 165	GCGTAGAGCGGCTGTTAGTGTCAAAT	GCGTTATCTCAAGTTTTGTAGCAG	A5
29	BARC 175	GCGTAACAGAAGCGGAGAAAGC	GCGAATCATTTAGTGTTAGGTGGC	A5-A6-D6
30	BARC 197	CGCATGGTCAGTTTTCTTTTAATC	GCGCTCTCCTTCATTTATGGTTTG	A3-A5-A4-B3
31	BARC 204	CGCAGAAGAAAAACCTCGCAGAAAA	CGCAGTGTATCCAAATGGGCAAG	A6 - D6
32	BARC 206	GCTTTGCCAGGTGAGCACTCT	TGGCCGGGTATTTGAGTTGGAGTT	A4-B3
33	BARC 216	CGCAGAAGAAAAACCTCGCAGAAAA	CGCAGTGTATCCAAATGGGCAAG	A6 - D6
34	BARC 240	AGAGGACGCTGAGAACTTTAGAGAA	GCGATCTTTGTAATGCATGGTGAA	A1-B1
35	CFA 152	TGGAAGTCTGGAACCACTCC	GCAACCAGACCACACTCTCA	D3
36	CFA 2040	TCAAATGATTTCAGGTAACCAC	TTCCTGATCCCACCAAACAT	7D,7A, 7B
37	CFA 2049	TAATTTGATTGGGTCGGAGC	CGTGTCGATGGTCTCCTTG	7A
38	CFA 2187	TAGCAAAGGGTGCATGTGAG	GCATGTTACGTCGCTGTTGT	A5,D1
39	CFA 2043	CAGCCGAAGAAGGATTTCTG	GAGGCAGGAACTTAGGGGAG	A2- B2
40	CFA 2070	TCTGAACCCTTGATTTTCCG	TTACTGGCAAGCCAGAACTGT	B5
41	CFA 2099	TGCGAAGTATTCAGTGCGTC	TCAAGACCATCAGCACTCAGA	A2/D7
42	CFA 2155	TTTGTTACAACCCAGGGGG	TTGTGTGGCGAAAGAAACAG	A5
43	CFA 2163	TTGATCCTTGATGGGAGGAG	CATCATTGTGTTTACGTTCTTTCA	A5
44	CFA 2185	TTCTTCAGTTGTTTTGGGGG	TTTGGTCGACAAGCAAATCA	A5
45	CFA 2190	CAGTCTGCAATCCACTTTGC	AAAAGGAAACTAAAGCGATGGA	A5
46	CFA 2256	GGTAATATTCAGGTTACCGCACA	GGTAAAGTTATAAATTGTTGTGGGC	A4
47	CFA 2	GGTTGCAGTTTCCACCTTGT	CATCTATTGCCAAAATCGCA	A4-A6-B1-D2-A3-D3-A4-B4-A5-B5-D5-D7
48	CFD 18	CATCCAACAGCACCAAGAGA	GCTACTACTATTTCATTGCGACCA	D5
49	CFD 49	TGAGTTCTTCTGGTGAGGCA	GAATCGGTTCACAAGGGAAA	6D
50	CFD 190	CAATCAGAAGCGCCATTGTT	CCCTGATGTTTTCTTTTTCTCC	A6-B6-D6
51	CFD 287	TCAAGAAGATGCGTTCATGC	GGGAGCTTTCCCTAGTGCTT	D6
52	GWM 160	TTCAATTCAGTCTTGGCTTGG	CTGCAGGAAAAAAAGTACACCC	4A
53	GWM 299	ACTACTTAGGCCTCCCGCC	TGACCCACTTGCAATTCATC	B3-B2
54	GWM 314	AGGAGCTCCTCTGTGCCAC	TTCGGGACTCTCTTCCCTG	3D
55	GWM 319	GGTTGCTGTACAAGTGTTCACG	CGGGTGCTGTGTGTAATGAC	B2
56	GWM 337	CCTCTTCCTCCCTCACTTAGC	TGCTAACTGGCCTTTGCC	B1-D1

57	GWM 340	GCAATCTTTTTTCTGACCACG	ACGAGGCAAGAACACACATG	3B
58	GWM 350	ACCTCATCCACATGTTCTACG	GCATGGATAGGACGCCC	4A
59	GWM 368	CCATTTCACCTAATGCCTGC	AATAAAACCATGAGCTCACTTGC	4B
60	GWM 382	GTCAGATAACGCCGTCCAAT	CTACGTGCACCACCATTTTG	2A,2B,2D
61	GWM 391	ATAGCGAAGTCTCCCTACTCCA	ATGTGCATGTCGGACGC	3A
62	GWM 413	TGCTTGTCTAGATTGCTTGGG	GATCGTCTCGTCCTTGGCA	1B
63	GWM 443	GGGTCTTCATCCGGAACTCT	CCATGATTTATAAATTCCACC	B5
64	GWM 493	TTCCCATAACTAAAACCGCG	GCAACATCATTTCTGGACTTTG	B3
65	GWM 497	GTAGTGAAGACAAGGGCATT	CCGAAAGTTGGGTGATATA	1A
66	GWM 501	GGCTATCTCTGGCGCTAAAA	TCCACAAACAAGTAGCGCC	2B
67	GWM 533	AAGGCGAATCAAACGGAATA	GTTGCTTTGGGGAAAAGC	3B,3D
68	WMC 42	GCCCTTGGTCCTGGGGTGAGCC	GCCTCATCCAGAGAGCCTGCGG	D7
69	WMC 99	AAGATGGACGTATGCATCACA	GCCATATTTGATGACGCATA	A1
70	WMC 114	ACAAACAGAAAATCAAAACCCG	ATCCATCGCCATTGGAGTG	B3-D3
71	WMC 166	ATAAAGCTGTCTCTTTAGTTCG	GTTTTAACACATATGCATACCT	A7-B7-D2-D7
72	WMC 210	TGCATCAAGAATAGTGTGGAAG	TGAGAGGAAGGCTCACACCT	B2-D2-A2
73	WMC 261	GATGTCATGTGAATCTCAAAAGTA	AAAGAGGGTCACAGAATAACCTAAA	A2-D1-B2-B3-B7
74	WMC 317	TGCTAGCAATGCTCCGGGTAAC	TCACGAAACCTTTTCCTCCTCC	2B
75	WMC 320	CATGCTTTCAAGACCTACGACA	GCAAACTTGCTCTGCTTGACTC	B1
76	WMC 329	ACAAAGGTGCATTCGTAGA	AACACGCATCAGTTTCAGT	1A,1B
77	WMC 333	TCAAGCATAGGTGGCTTCGG	ACAGCAGCCTTCAAGCGTTC	1A
78	WMC 336	GTCTTACCCCGCGATCTGC	GCGGCCTGAGCTTCTTGAG	1D
79	WMC 356	GCCGTTGCCCAATGTAGAAG	CCAGAGAAACTCGCCGTGTC	2B
80	WMC 361	CTTCAGAGATGTTTGATTACCT	GGTATTGCTAACTGAATGATGT	B7-D4
81	WMC 406	TATGAGGGTCGGATCAATACAA	CGAGTTTACTGCAAACAAATGG	B1
82	WMC 413	CACTGGAAACATCTCTTCAACT	ACAGGAAAGGATGATGTTCTCT	4B
83	WMC 420	ATCGTCAACAAAATCTGAAGTG	TTACTTTTGCTGAGAAAACCCT	4A
84	WMC 435	GCACTATACTTATTGGATTGTCA	CATGGTATCCCTAGTAAGTTTTT	B2-D3-B5-B7
85	WMC 468	AGCTGGGTTAATAACAGAGGAT	CACATAACTGTCCACTCCTTTC	A4
86	WMC 500	ATAGCATGTTGGAACAGAGCAC	CTTAGATGCAACTCTATGCGGT	B1-B2-B3-B5-B7-A4
87	WMC 524	TAGTCCACCGGACGGAAAGTAT	GTACCACCGATTGATGCTTGAG	5A
88	WMC 532	GATACATCAAGATCGTGCCAAA	GGGAGAAATCATTAACGAAGGG	3A
89	WMC 553	CGGAGCATGCAGCTAGTAA	CGCCTGCAGAATTCAACAC	A4
90	WMC 580	AAGGCGCACAACACAATGAC	GGTCTTTTGTGCAGTGAACTGAAG	A6
91	WMC 617	CCACTAGGAAGAAGGGGAAACT	ATCTGGATTACTGGCCAACTGT	4A
92	WMC 687	AGGACGCCTGAATCCGAG	GGGAGCGTAGGAGGACTAACA	3B
93	WMC 765	GGGATCAGACTGGGACTGGAG	GGGTTGGCTTGGCAGAGAA	5D
94	WMC 805	GATGCTGCTGCACCAAACTC	GCCTTTTCCATGCCACACT	5A
95	WMC 807	ATCCAACAAGGCCTCACCAT	GCAGGTTTGATCTGGATTTCATC	6A
96	WMC 810	GGCACCGATGCTTCCA	GCCCCAACCACCTCCC	5B
97	WMC 173	CATGGTGGCCATGAGTGGAGGT	CATGATCTTGCGTGTGCGTAGG	D1-B2-A6-B6-A7-A2-A4
98	WMS 5	GCCAGCTACCTCGATACAACTC	AGAAAGGGCCAGGCTAGTAGT	A3
99	WMS 10	CGCACCATCTGTATCATTCTG	TGGTCGTACCAAAGTATACGG	A2-B2-A3-A7
100	WMS 24	CACACAAGGCACCATTGC	CAATGGACATAGTTGTGTGCG	D2-B1
101	WMS 44	GTTGAGCTTTTCAGTTCGGC	ACTGGCATCCACTGAGCTG	D7-A4
102	WMS 46	GCACGTGAATGGATTGGAC	TGACCCAATAGTGGTGGTCA	B7
103	WMS 52	CTATGAGGCGGAGGTTGAAG	TGCGGTGCTCTTCCATTT	D3
104	WMS 55	GCATCTGGTACACTAGCTGCC	TCATGGATGCATCACATCCT	B2-D6
105	WMS 58	TCTGATCCCGTGAGTGTAACA	GAAAAAAATTGCATATGAGCCC	B6
106	WMS 63	TCGACCTGATCGCCCCTA	CGCCCTGGGTGATGAATAGT	A7
107	WMS 67	ACCACACAAACAAGGTAAGCG	CAACCCTCTTAATTTTGTTGGG	A3-B5
108	WMS 72	TGGTCCCTCTCCCTTTCTCT	ACAGAATTGAAGATTGTCGGTC	B3
109	WMS77	ACAAAGGTAAGCAGCACCTG	ACCCTCTTGCCCGTGTTG	B3
110	WMS 107	ATTAATACCTGAGGGAGGTGC	GGTCTCAGGAGCAAGAACAC	B4-B3-B6
111	WMS 118	GATGTTGCCACTTGAGCATG	GATTAGTCAAATGGAACACCCC	A4-B5
112	WMS 124	GCCATGGCTATCACCCAG	ACTGTTCGGTGCAATTTGAG	B1
113	WMS 148	GTGAGGCAGCAAGAGAGAAA	CAAAGCTTGACTCAGACCAAA	B2
114	WMS 155	CAATCATTTCCCCCTCCC	AATCATTGGAAATCCATATGCC	A3
115	WMS 189	AGGAGCAGCGGAACGAAC	AGAAATACGGAAACCCACCC	B2
116	WMS 190	GTGCTTGCTGAGCTATGAGTC	GTGCCACGTGGTACCTTTG	D5
117	WMS 297	ATCGTCACGTATTTTGCAATG	TGCGTAAGTCTAGCATTTTCTG	7B
118	WMS 403	CGACATTGGCTTCGGTG	ATAAAACAGTGCGGTCCAGG	1B

119	WMS 493	TTCCCATAACTAAAACCGCG	GGAACATCATTTCTGGACTTTG	3B
120	WMS 566	TCTGTCTACCCATGGGATTTG	CTGGCTTCGAGGTAAGCAAC	3B
